# Association of Sedentary and Active Bout Frequency With Mortality in Older Men Using Accelerometry

**DOI:** 10.1093/geroni/igab046.3467

**Published:** 2021-12-17

**Authors:** Lauren S Roe, Stephanie Harrison, Peggy Cawthon, Kyle Moored, Kristine Ensrud, Katie Stone, Kelley Pettee Gabriel, Jane Cauley

**Affiliations:** 1 University of Pittsburgh, Pittsburgh, Pennsylvania, United States; 2 California Pacific Medical Center, Research Institute, San Francisco, California, United States; 3 University of Minnesota, Minneapolis, Minnesota, United States; 4 University of California, Berkley, San Francisco, California, United States; 5 The University of Alabama at Birmingham, Birmingham, Alabama, United States

## Abstract

BACKGROUND: Time spent sedentary increases with age and has several negative health consequences. We sought to examine associations between daily sedentary and active bout frequency with all-cause mortality. METHODS: Data are from 2,918 men in the Osteoporotic Fractures in Men (MrOS) study (mean age at Visit 3±SD: 79.0±5.1 years) with valid activity monitor data (5.1±0.3 days worn>90%) at Year 7 visit (Visit 3, 2007-2009). Sedentary and active bout frequencies are defined as the daily transition frequency from a sedentary bout lasting 5+ minutes to activity of any intensity, and the transition frequency from an active bout lasting 5+ minutes to sedentary. Deaths were centrally adjudicated using death certificates. Cox proportional hazard models were used to examine associations between quartiles of sedentary (Q1 referent, <13.6 bouts/day) or active (Q1 referent, <5 bouts/day) bout frequency and mortality. Models were repeated, stratifying by median daily total time spent sedentary and active. RESULTS: After 9.4±3.7 years of follow-up, 1,487 (51.0%) men died. Men averaged 16.9±5.1 and 8.2±4.2 sedentary and active bouts/day, respectively. After full covariate adjustment, each quartile reflecting a higher sedentary (Q4 vs Q1 HR: 0.68, 95%CI: 0.58-0.81, p-trend<0.001) and active bout (Q4 vs Q1 HR: 0.57, 95%CI: 0.48-0.68, p-trend<0.001) frequency was associated with lower mortality risk. There was no evidence that effects differed by total sedentary time (p-interaction for sedentary bout frequency and total sedentary time>0.05). CONCLUSIONS: More frequent, prolonged sedentary and active bouts are associated with a lower mortality risk in older men and is not moderated by total sedentary time.

